# Prolonged infusion of meropenem for suspected intracystic infection in polycystic kidney disease during pregnancy: a case report

**DOI:** 10.3389/fmed.2026.1856558

**Published:** 2026-10-05

**Authors:** Bingqing Ruan, Danjia Xie, Liangqi Yan, Nianying Deng

**Affiliations:** 1 Department of Internal Medicine, Wenling Maternal and Child Health Hospital, Wenling, Zhejiang, China; 2 Ultrasound Room, Wenling Maternal and Child Health Hospital, Wenling, Zhejiang, China; 3 Department of Pharmacy, Wenling Maternal and Child Health Hospital, Wenling, Zhejiang, China

**Keywords:** intracystic infection, meropenem, polycystic kidney disease, pregnancy, prolonged infusion

## Abstract

**Background:**

Intrarenal cyst infection in polycystic kidney disease (PKD) is hard to confirm and harder to treat during pregnancy, when lipid-soluble antibiotics are generally avoided.

**Case:**

A pregnant woman at approximately 10 weeks’ gestation presented with recurrent fever and flank pain for over one month, with acute exacerbation one day prior to admission. Laboratory tests revealed progressively elevated C-reactive protein (CRP) levels despite repeatedly negative cultures. Serial ultrasound showed a right upper-pole renal cyst that evolved to exhibit internal septations and debris.

**Treatment and outcome:**

Cyst aspiration was avoided; meropenem 1 g every 8 h was initiated on admission and converted to prolonged infusion (1 g infused over more than 3 h) on Day 4, followed by step-down oral amoxicillin–clavulanate, for a total antimicrobial course of 32 days. Fever resolved and CRP normalized; pregnancy continued uneventfully.

**Conclusion:**

In a pregnant patient with suspected PKD cyst infection, prolonged-infusion meropenem was followed by clinical improvement. The favorable response observed supports further evaluation of this therapeutic strategy, despite the absence of microbiological confirmation.

## Introduction

Polycystic kidney disease (PKD) is a common inherited renal disorder characterized by progressive cyst formation and enlargement in both kidneys. Infectious complications, particularly intracystic infections, represent a major cause of morbidity and are often difficult to diagnose and manage. Clinical presentation is frequently nonspecific; microbiological confirmation is uncommon, and imaging findings may be subtle or delayed (1, 2). These challenges are further amplified during pregnancy, when physiological changes increase susceptibility to urinary tract infections and when diagnostic and therapeutic options are restricted to protect fetal safety (3, 4).

Management of cyst infections in pregnancy is especially complex. Many antimicrobial agents with favorable cyst penetration are generally avoided in pregnancy because of fetal safety concerns that differ by agent and gestational period, and those antibiotics considered safe in pregnancy often demonstrate limited penetration into cyst fluid (2, 5). As a result, treatment decisions are commonly guided by indirect evidence, pharmacokinetic considerations, and individualized risk assessment rather than robust clinical trial data (3, 6).

The purpose of this case report is to describe the diagnosis and management of a suspected intracystic infection in a pregnant patient with PKD treated with prolonged infusion of meropenem (MEM).

## Case presentation

A 22-year-old gravida 1 para 0 woman at approximately 10 weeks of gestation presented to our hospital with a one-month history of recurrent fever and right flank pain, with worsening symptoms for one day prior to admission. She had a history of PKD, which was diagnosed prior to pregnancy. There was no history of recent urinary tract instrumentation or trauma.

On admission, analysis of her vital signs revealed a temperature of up to 38.8 °C, heart rate of 102 beats/min, and blood pressure of 116/77 mmHg. Physical examination was notable for right costovertebral angle tenderness without overt peritoneal signs. Initial laboratory studies demonstrated leukocytosis with neutrophilia, elevated inflammatory markers including C-reactive protein (CRP) and procalcitonin (PCT), and pyuria. On admission, two sets of blood cultures, each comprising paired aerobic and anaerobic bottles, were drawn from separate venipunctures before the first dose of meropenem, and clean-catch midstream urine specimens were obtained before antibiotic administration and repeated on April 13, 2025; significant bacteriuria was defined as ≥10⁵ CFU/mL for gram-negative bacteria and ≥10⁴ CFU/mL for gram-positive bacteria. All cultures obtained during this admission remained negative. Approximately five weeks before the current admission, the patient had been hospitalized at an outside facility (February 27 to March 6, 2025) for fever and right flank pain. During that admission she received intravenous cefoperazone-sulbactam 1.5 g twice daily from February 27 to March 2, with no clinical improvement; therapy was escalated to intravenous meropenem 0.5 g every 8 h (standard infusion) from March 3 to March 6, after which her fever began to resolve on March 4. A urine culture obtained on February 28 grew Escherichia coli producing extended-spectrum beta-lactamase (ESBL), reported as sensitive to cefoperazone-sulbactam (MIC less than or equal to 8 mcg/mL). Renal ultrasonography at that time showed bilateral polycystic kidneys without evidence of complex cysts, internal septations, or debris. The patient remained off all antibiotics for more than one month before the current admission. Given this prolonged antibiotic-free interval, the repeatedly negative blood and urine cultures obtained during the current hospitalization are more likely attributable to the walled-off, poorly communicating nature of the infected cyst than to antibiotic carryover. Blood pressure remained normotensive throughout hospitalization, ranging from 100 to 126 mmHg systolic and 64 to 77 mmHg diastolic, with no recorded value meeting the threshold for hypertension in pregnancy (systolic at least 140 mmHg or diastolic at least 90 mmHg).

Ultrasonography on April 11 showed bilateral renal cysts consistent with known PKD, with multiple anechoic areas within both renal parenchyma (Figure 1A). The right kidney measured approximately 150 mm × 70 mm, the left kidney was approximately 149 mm × 71 mm, and the collecting systems were not dilated. No focal lesion suggestive of infection was definitively seen at that time.

**Figure 1:**
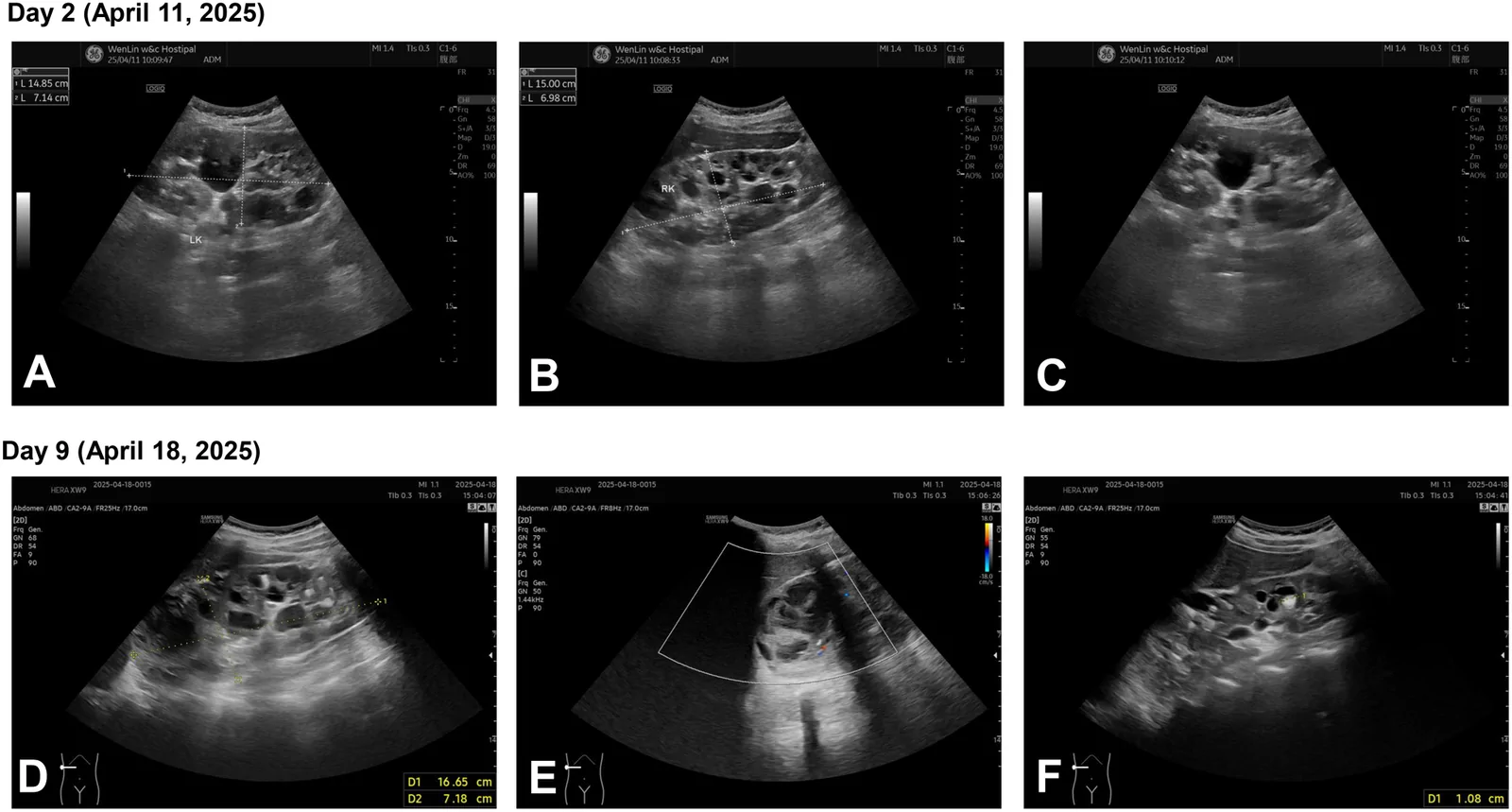
Serial renal ultrasonography in a pregnant woman with polycystic kidney disease (PKD). **(A–C)** Day 2 (April 11, 2025) baseline examination. Both kidneys are enlarged with multiple simple anechoic cysts and an indistinct corticomedullary junction (A, left kidney; B, right kidney; C, left renal cyst close-up). On panels A and B, the machine-generated annotations “LK” and “RK” identify the left and right kidneys, respectively, while “L” within the measurement data (e.g., “1 L 15.00 cm”) denotes the length measurement, not a laterality marker. No sonographic features suggestive of intracystic infection (complex cyst, wall thickening, internal debris, or septations) are observed, and no collecting system dilatation or ureterectasis is identified. **(D–F)** Day 9 (April 18, 2025) follow-up examination after initiation of prolonged-infusion meropenem. The right kidney demonstrates a complex cystic lesion in the upper pole (panels D, E), reported as approximately 5.48 × 4.63 cm, with internal septations, low-level internal echoes or debris, and reduced through-transmission. Echogenic foci with posterior acoustic shadowing consistent with nephrolithiasis are also present (panel F). In the appropriate clinical context, these evolving sonographic findings support suspected intracystic infection in the right kidney.

Despite the antimicrobial therapy administered during the prior hospitalization, the patient re-presented with recurrent fever and worsening flank pain, indicating that the earlier treatment course had not eradicated the source of infection and that a more intensive approach was warranted. Repeat ultrasonography on April 18 revealed increased complexity of cystic structures in the right upper renal pole, including a 55 mm × 46 mm liquid dark area with internal septations and reduced echogenicity, highly suggestive of a complicated cyst or suspected intracystic infection (Figure 1B).

At the current admission, meropenem 1 g every 8 h was initiated on the day of admission (Day 2) as first-line therapy, and no other antibiotics were administered during the current admission before its initiation. Renal function was preserved both before admission [serum creatinine 53 µmol/L [0.60 mg/dL], eGFR 129 mL/min/1.73 m^2^ on March 31, 2025; serum creatinine 44 µmol/L [0.50 mg/dL], eGFR 134 mL/min/1.73 m^2^ on Day 2; and serum creatinine 38 µmol/L [0.43 mg/dL], eGFR 139 mL/min/1.73 m^2^ on Day 5]with no evidence of acute kidney injury. eGFR was estimated with the CKD-EPI (2021) equation; as this equation was validated in non-pregnant populations and pregnancy physiologically lowers serum creatinine, the true GFR may be somewhat higher than estimated, and these values were interpreted accordingly. The preserved renal function supported the use of the standard meropenem regimen (1 g every 8 h) without dose adjustment. Other relevant findings included absence of hydronephrosis and normal bladder filling. Obstetric ultrasound at the same gestational age showed a viable intrauterine pregnancy with appropriate fetal size and amniotic fluid volume, without overt anomaly.

Fetal monitoring during hospitalization. Serial obstetric ultrasonography was performed throughout the admission. On April 12, 2025 (approximately 10 weeks of gestation, during the febrile period and while receiving meropenem treatment), a viable intrauterine pregnancy was confirmed with a crown-rump length of 35 mm, consistent with gestational age; fetal heart rate and fetal movements were visualized and judged to be appropriate. On April 22, 2025 (approximately 11 weeks of gestation), a follow-up ultrasound showed a crown-rump length of 52 mm, a biparietal diameter of 17 mm, visible fetal movements, a fetal heart rate of 191 beats per minute, and a maximum amniotic fluid pocket of 30 mm. The fetal heart rate of 191 beats per minute, although at the upper end of the physiologic range for 11 weeks of gestation, was likely influenced in part by the concurrent maternal pyrexia (peak temperature 39.6 to 40.0 degrees Celsius). Bilateral uterine artery Doppler examination on April 23, 2025 showed flow indices within the normal range, with no evidence of increased uteroplacental resistance. On May 1, 2025 (approximately 12 weeks of gestation, after completion of intravenous meropenem and transition to oral amoxicillin-clavulanate), a first-trimester screening ultrasound demonstrated a fetal heart rate of 167 beats per minute, which had normalized following maternal defervescence, together with a crown-rump length of 66 mm, a biparietal diameter of 23 mm, a head circumference of 81 mm, an abdominal circumference of 68 mm, a femur length of 8 mm, a nuchal translucency of 1.6 mm, a maximum amniotic fluid pocket of 40 mm, visible fetal movements, a present nasal bone, and a ductus venosus waveform without a-wave reversal. All growth parameters were consistent with gestational age. At no point during hospitalization did the patient report a subjective decrease in fetal movements.

## Treatment and outcome

Upon admission, broad-spectrum antimicrobial therapy was initiated and optimized in consultation with the clinical pharmacy team. Considering the patient's pregnancy and the suspected diagnosis of intracystic infection in PKD, antibiotic options were limited because of fetal safety concerns (fluoroquinolones and trimethoprim-sulfamethoxazole are generally avoided in pregnancy) and the poor cyst penetration of many agents. Empirical meropenem was chosen because a urine culture obtained during the prior hospitalization had grown ESBL-producing Escherichia coli, for which carbapenems are the preferred agents given the high failure rates of cephalosporins (7); because the same patient had already failed cefoperazone-sulbactam clinically despite reported *in vitro* susceptibility; and because meropenem has the most extensive clinical experience among carbapenems in pregnancy (8). National surveillance data confirm that ESBL-producing E. coli remain highly prevalent in China (9), supporting empirical carbapenem coverage in this setting. Narrower pregnancy-compatible beta-lactam alternatives (e.g., piperacillin-tazobactam, cefepime) were considered, but the documented ESBL-producing organism and prior treatment failure favored a carbapenem. MEM 1 g every 8 h was initiated on the day of admission (Day 2) as a standard infusion and converted to prolonged infusion (1 g infused over more than 3 h) on Day 4. The prolonged-infusion strategy was chosen as a theoretically plausible way to optimize time-dependent pharmacodynamics, maximizing the time above the minimum inhibitory concentration (fT > MIC) in an effort to improve activity within cystic lesions.

Adjunctive supportive measures including antipyretics, hydration, and fetal-friendly anti-nausea therapy were also provided. Low molecular weight heparin was administered for thromboprophylaxis in the setting of pregnancy and immobility (10).

Serial monitoring of clinical parameters and laboratory markers demonstrated an initial improvement in inflammatory indices (CRP and PCT) and reduction in leukocytosis over the ensuing 7–10 days. However, episodes of fever persisted intermittently, consistent with ongoing localized cystic involvement rather than systemic bacteremia. Urine and blood cultures remained negative throughout the hospitalization.

The patient's clinical symptoms gradually improved during the inpatient course (Day 2 to Day 10, April 11 to 19, 2025), and she was discharged on Day 10 on orally administered amoxicillin-clavulanate 312.5 mg three times daily for continued sequential therapy, with instructions for close outpatient follow-up. Amoxicillin-clavulanate was chosen because oral options compatible with the first trimester are very limited and it is compatible with pregnancy, achieving high concentrations in urine; it was prescribed for 23 days (April 20 to May 12, 2025), completing the total antimicrobial course of 32 days (intravenous meropenem for 9 days, April 11 to 19, followed by oral amoxicillin-clavulanate for 23 days, April 20 to May 12). A detailed timeline of key clinical events, laboratory responses, and imaging findings is summarized in Figure 2, and the inflammatory marker trends are presented in Table 1.

**Figure 2:**
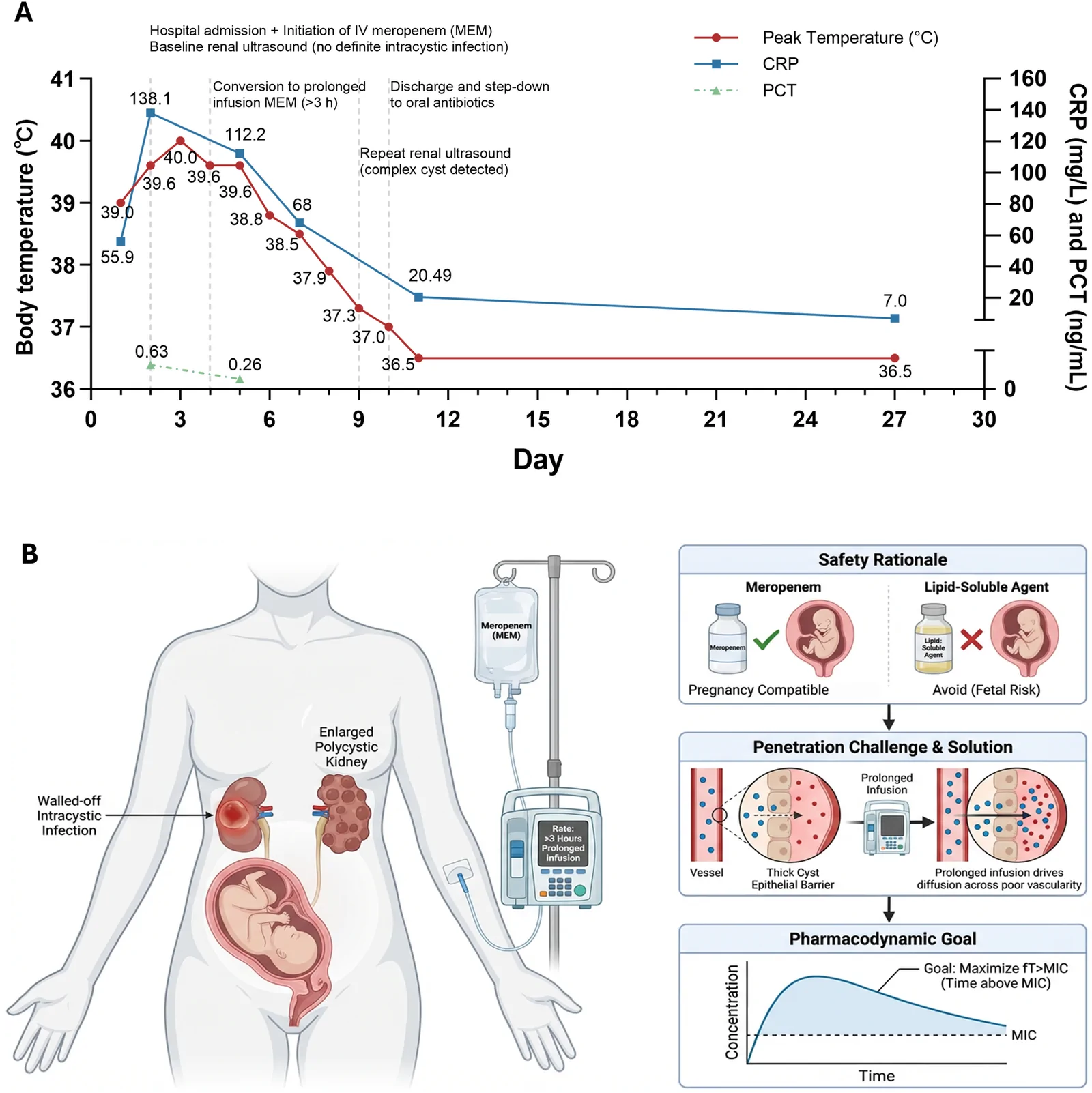
Chronological clinical course and proposed mechanism-based management of suspected intracystic infection in PKD during early pregnancy. **(A)** Body temperature is plotted from symptom onset to discharge and follow-up. Persistent high-grade fever is shown before admission. Gradual defervescence is shown after meropenem (MEM) is administered by prolonged infusion. C-reactive protein (CRP) and procalcitonin (PCT) show a marked decline following antimicrobial escalation, although fever recurs intermittently. Key diagnostic and therapeutic milestones are annotated, including hospital admission, initiation and escalation of MEM, conversion to prolonged infusion on Day 4, and step-down to oral antibiotics at discharge. Renal ultrasonography time points: Imaging on Day 2 shows bilateral polycystic kidneys without definite signs of intracystic infection; and repeat imaging on Day 9 shows a complex cystic lesion in the right upper pole with internal septations and poor acoustic transmission, supporting suspected intracystic infection. US: ultrasound. **(B)** A simplified mechanism and treatment rationale are illustrated. Bacterial seeding is presumed to occur via ascending urinary tract infection and hematogenous spread. A walled-off infection is established within a cyst. Immune access and antibiotic penetration are limited by the cyst epithelial barrier and poor vascularity. Sterile cultures and delayed radiologic evolution are therefore possible. In the first trimester, lipid-soluble agents with superior cyst penetration are avoided. MEM is selected as a pregnancy-compatible broad-spectrum alternative. Prolonged infusion (>3 h per dose) is used to increase fT > MIC and support diffusion-driven activity within the infected cyst. This image was generated using Nano Banana Pro but underwent strict human review and modification by author.

**Table 1:** Serial changes in infection and inflammatory parameters during hospitalization and follow-up.

Day	Peak Temperature ( °C)	WBC ( × 10⁹/L)	Neutrophils ( × 10⁹/L or %)	CRP (mg/L)	PCT (ng/mL)	Urinalysis (RBC/WBC/Protein)	Serum creatinine (µmol/L)	eGFR (mL/min/1.73 m^2^)	Hemoglobin
Day 1 (Febrile, Pre-admission)	39.0	12.87	7.9	55.9	/	RBC 2+, WBC 3+, Protein 2+	/	/	106
Day 2 (Admission, MEM iv)	39.6	14.2	9.8	138.1	0.63	RBC 4+, WBC 1+, Protein 2+	44	134	101
Day 3 (MEM 1 g Q8h)	40	/	/	/	/	/	/	/	/
Day 4 (MEM prolonged infusion)	39.6	/	/	/	/	/	/	/	/
Day 5	39.6	9.1	5.0	112.2	0.26	RBC 2+, WBC –, Protein +	38	139	99
Day 6	38.8	/	/	/	/	/	/	/	/
Day 7	38.5	6.8	2.7	68.0	/	RBC –, WBC +, Protein +	/	/	86
Day 8	37.9	/	/	/	/	/	/	/	/
Day 9	37.3	/	/	/	/	/	/	/	/
Day 10 (Discharge)	37.0	/	/	/	/	/	/	/	/
Day 11 (Follow-up)	36.5	10.49	4.69	20.49	/	RBC –, WBC –, Protein +	/	/	101
Day 27 (Follow-up)	36.5	9.0	4.4	7.0	/	RBC –, WBC –, Protein –	/	/	100

MEM, meropenem; WBC, white blood cell count; CRP, C-reactive protein; PCT, procalcitonin; PKD, polycystic kidney disease; US, ultrasonography. MEM was initiated on Day 2 at 1 g every 8 h. The prolonged infusion of MEM (1 g over more than 3 h per dose, using an infusion pump) was started on Day 4. Blood and urine cultures remained negative throughout hospitalization. eGFR was estimated using the CKD-EPI 2021 equation; values in pregnancy should be interpreted with caution as the equation may underestimate true GFR.

Post-discharge follow-up via outpatient visits and telephone communication confirmed continued resolution of symptoms, normalization of inflammatory markers, and no recurrent fever; blood pressure remained normal throughout all subsequent prenatal checkups, with no evidence of gestational hypertension. A follow-up urinalysis on May 6, 2025 (Day 27) was normal. Repeat renal imaging was deferred because of clinical improvement and pregnancy status, with plans for postnatal assessment. The pregnancy continued with routine prenatal care. The patient delivered at 39 weeks of gestation by cesarean section. The infant was liveborn with a birth weight of approximately 3,100 g, appropriate for gestational age, and the Apgar scores were 9 and 10 at 1 and 5 min, respectively. No gross congenital anomalies were identified on neonatal examination. The birth weight is approximate, as it was obtained through telephone follow-up after the patient returned to her hometown for delivery. Given the limited duration of postnatal follow-up available at the time of writing, long-term growth and neurodevelopmental data for the infant are not yet available.

## Discussion

PKD is associated with significant morbidity from cyst infections, which represent one of the most challenging complications because of their insidious onset, nonspecific presentation, and frequent lack of microbiological confirmation (1, 11). In non-pregnant patients, cyst aspiration remains the diagnostic gold standard; however, even in these cases, cultures are often negative because of the isolated nature of the cystic compartment (2). During pregnancy, these difficulties are compounded by the need to avoid invasive procedures and ionizing radiation, forcing reliance on indirect criteria: persistent fever, localized flank pain, elevated inflammatory markers, exclusion of alternative foci, and serial ultrasonographic changes suggestive of cyst complexity (3, 6). In the present case, the evolution from an initially unremarkable ultrasound to a right upper-pole cyst demonstrating internal septations and debris over one week provided critical supportive evidence in the absence of more advanced imaging modalities. Pregnancy itself confers additional risk through physiologic urinary stasis, progesterone-mediated ureteral dilation, and relative immunosuppression, all of which may facilitate ascending infection or hematogenous seeding of cysts (3, 4). Pyelonephritis, hemorrhagic cyst, and renal abscess could not be entirely excluded and remained in the differential diagnosis. Nevertheless, several features favored a suspected intracystic infection: the pain was strictly right-sided with localized costovertebral angle tenderness, serial ultrasound showed no collecting-system dilatation or ureterectasis, and a single right upper-pole cyst evolved over one week into a complex lesion with internal septations and debris. The repeatedly negative blood and urine cultures cannot, on their own, exclude pyelonephritis or low-grade bacteremia. However, because the patient had been antibiotic-free for over one month before these cultures were obtained and all cultures were drawn before antimicrobial administration during the current admission, the negative results are more consistent with sequestration of infection within a poorly communicating cyst than with antibiotic suppression; they are therefore regarded as supportive rather than decisive evidence, particularly because prior antibiotic exposure at the outside facility could theoretically have reduced culture yield. The trajectory of inflammatory markers (CRP peaking at 138.1 mg/L and PCT at 0.63 ng/mL, followed by a slow decline over 7 to 10 days) further supported a walled-off, poorly drained focus rather than a freely communicating urinary tract infection, as did the clinical response pattern of persistent low-grade fever despite initial broad-spectrum coverage, with gradual resolution observed during prolonged-infusion meropenem therapy. Hemorrhagic cysts were also considered, but the prolonged febrile course and progressive inflammatory response made suspected intracystic infection more probable.

Lipid-soluble antibiotics, including fluoroquinolones and trimethoprim-sulfamethoxazole, are favored by current guidelines and expert consensus because of their reliable penetration into cyst fluid (2, 5). However, fluoroquinolones are generally avoided in pregnancy because of concerns about fetal cartilage and arthropathy derived from animal studies and the lack of established human safety data, whereas trimethoprim-sulfamethoxazole is a folate antagonist whose first-trimester use has been associated with neural tube defects in some studies and which is avoided near term because of the risk of kernicterus; both were therefore avoided in this first-trimester patient, leaving few viable alternatives (12). Beta-lactams are generally regarded as safe in pregnancy; meropenem has not been shown to be teratogenic in animal reproduction studies, but adequate and well-controlled human data in the first trimester are lacking, and available reports have not shown a consistent pattern of adverse fetal outcomes (12). Beta-lactams achieve substantially lower intracystic concentrations (13). MEM, a broad-spectrum carbapenem, was selected here for its favorable fetal safety profile and activity against common urinary pathogens. Prolonged infusion is a theoretically plausible strategy to maximize the time above the minimum inhibitory concentration (fT > MIC) for this time-dependent beta-lactam and to offset its limited penetration into infected cysts (13). Direct evidence that prolonged infusion improves intracystic meropenem concentrations is lacking: the pharmacokinetic study by Hamanoue et al. (13) documented poor meropenem penetration into infected cysts but did not compare standard and prolonged infusion regimens, and no organism, MIC, or drug concentration measurements were available in the present case. Because the patient had also received meropenem and other antibiotics before conversion to prolonged infusion, the observed clinical improvement cannot be attributed specifically to the prolonged-infusion strategy. The gradual decline in CRP, procalcitonin, and leukocytosis over 7–10 days, despite intermittent low-grade fever, was consistent with the slow resolution expected in a walled-off infectious focus, although the specific contribution of the prolonged-infusion strategy could not be established.

In the current patient, supportive measures were tailored to the pregnant state. Low-molecular-weight heparin was administered for venous thromboembolism prophylaxis, given the synergistic risks of pregnancy, systemic inflammation, and prolonged hospitalization (10). Sequential therapy with oral amoxicillin-clavulanate upon discharge facilitated early mobilization while maintaining coverage; however, because the susceptibility of the presumed pathogen was not established and ESBL-producing E. coli are frequently resistant to amoxicillin-clavulanate, the oral step-down is best regarded as pragmatic consolidation therapy rather than demonstrated cure. Amniocentesis later confirmed fetal inheritance of the pathogenic PKD variant, highlighting the importance of pre-conception counseling in affected women. This finding was anticipated given the mother's diagnosis and does not represent an adverse effect of either the infection or its treatment.

## Conclusion

Clinical improvement was observed after prolonged-infusion meropenem in a pregnant patient with suspected ADPKD cyst infection. While microbiological confirmation was not obtained, the diagnosis was supported by a consistent composite of clinical, laboratory, and imaging findings; within this diagnostic framework, the favorable response observed warrants further evaluation of prolonged-infusion meropenem as a therapeutic option for this challenging condition.

## Patient perspective

At the time of admission, the pregnant woman was strongly motivated to continue the pregnancy. It was clearly expressed that the child was wished to be carried to term regardless of whether the PKD-associated mutation was present. Throughout the clinical course, active cooperation with medical decision-making was maintained, with a consistently positive and determined attitude toward both treatment and pregnancy outcome.

## Data Availability

The original contributions presented in the study are included in the article/Supplementary Material, further inquiries can be directed to the corresponding author.
